# High-performance dye-sensitized solar cells using Ag-doped CoS counter electrodes†

**DOI:** 10.1039/c8ra02765j

**Published:** 2018-05-22

**Authors:** Guoce Zhuang, Huiling Liu, Xiaobo Chen

**Affiliations:** School of New Energy and Electronic Engineering, Yancheng Teachers University Yancheng 224051 P. R. China chenxbok@126.com +86-515-8823-3177

## Abstract

CoS has been emerging as a promising Pt-free counter electrode (CE) material for dye-sensitized solar cells (DSSCs) due to its satisfactory electrocatalytic properties for redox reactions. However, its low electronic and ionic conductivities have limited its use in DSSCs. The doping of Ag with appropriate amount significantly improved the properties of CoS for application as a CE. Ag-doped CoS samples with various doping amounts were prepared by a facile one-step hydrothermal approach. There were very sharp changes of morphologies and particle sizes after doping different amounts of Ag. It is found that the DSSC fabricated with the 5% Ag-doped CoS CE achieved an impressive power-conversion efficiency (PCE) of 8.35% which was higher than that of the DSSC with a Pt CE (8.17%) by 2.2%, while the DSSC consisting of undoped CoS only exhibited a PCE of 6.93%. Such an enhanced PCE could be attributed to the significantly improved electrochemical activity and mixed conductivity resulting from the Ag dopant. Therefore, the excellent electrocatalytic activity, facile preparation and low material cost of the Ag-doped CoS electrode provide it with promising potential for large-scale production of new-generation DSSCs.

## Abstract 2

The PCE of 5% Ag-doped CoS based solar cells is 20.5% higher than that of solar cells based on un-doped CoS.

## Graphical abstract

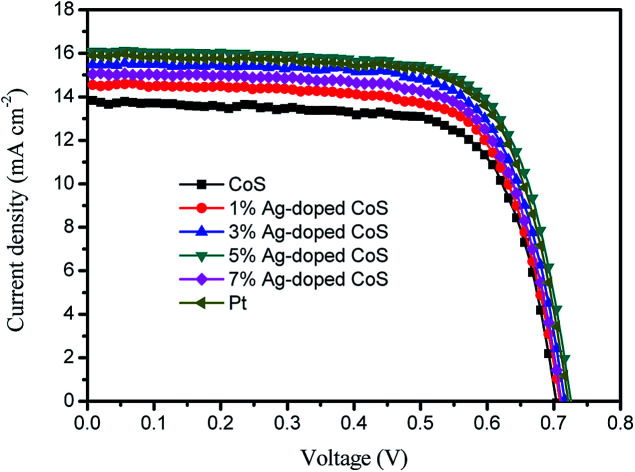

## 1. Introduction

Dye-sensitized solar cells (DSSCs) have attracted much attention as an alternative to silicon-based solar cells due to their low cost, easy fabrication methods and eco-friendly nature.^1–3^ As a critical component of DSSCs, the counter electrode (CE) has a great influence on the reduction of I_3_^−^/I^−^ and conducts electrons from the external circuit to the cell.^4–6^ Normally, platinum (Pt) is used as the CE material in high efficiency DSSCs because of its excellent electrocatalytic activity and high electrical conductivity.^7^ However, the use of Pt, a noble metal, hinders the large-scale application of DSSCs due to its relatively high cost. Recently, intensive research efforts have been made to investigate different counter electrode materials, including various carbon allotrope materials,^8,9^ conductive polymers,^10,11^ metal carbides,^12,13^ nitrides,^14,15^ oxides^16,17^ and sulfides.^18,19^

The electrochemical process at the CE mainly comprises two steps: redox reactions at the electrode–electrolyte interface, and charge and mass transfer through the electrode. Cobaltous sulfide (CoS and CoS_2_) has been widely investigated for applications in electrochemical energy storage,^20,21^ photocatalysis^22,23^ and DSSCs,^24,25^ because of their environmental friendliness, low cost of production and excellent electrocatalytic activity. The PCE of DSSCs based on pure CoS CEs is usually incomparable to DSSCs consisting of Pt CEs,^26,27^ due to their relatively low electrical conductivity and limited ion diffusion rate. Therefore, modification of CoS such as impurity doping^28^ and composite with carbon materials^26^ is required to enhance its performance as an efficient CE in DSSCs.

Among various modification methods, impurity doping has been recognized as a cost-effective strategy to tune the physical and chemical properties of metal oxides and sulfides and even to optimize their crystal structures and morphologies by introducing defects into the materials. It is published that the participation of the introduced defects can increase the surface energy and reduce electrostatic repulsion between adjacent layers, thereby eventually changing the migration energy and diffusion barriers.^29^ Recently, Ag-doped metal sulfide materials have observed significantly enhanced electronic conductivity and electrochemical properties.^30,31^ Doping Ag into SnS_2_ CE material for DSSC could greatly improve the electrocatalytic activity and mixed ionic–electronic conductivity which was reported by Wang' group.^30^ Motivated by these advances, it is reasonable to expect a similar influence of Ag doping on the performance of DSSCs with CoS CEs.

In this work, Ag-doped CoS nanostructures as CEs in DSSCs are synthesized *via* a facile one-step hydrothermal method for the first time. The effects of Ag-doping concentration on the crystal structure, morphology and chemical bonding of CoS are symmetrically investigated. The PCE of DSSCs using Ag-doped CoS is optimized by varying the Ag doping amount. The Ag doping into CoS can effectively improve both the electrocatalytic activity and mixed ionic–electronic conductivity. Benefiting from the superior catalytic performance, the 5% Ag-doped CoS CE exhibits superior PCE of 8.35%, which is comparable or superior to many of the reported CoS based DSSCs (Table S1†).

## 2. Experimental

### 2.1. Ag-doped CoS synthesis

Ag-doped CoS nanostructures were prepared *via* a hydrothermal method. CoCl_2_·6H_2_O (0.1903 g), CH_4_N_2_S (0.1218 g), and AgSO_4_ were dissolved in 10 mL of absolute ethanol with vigorous agitation. The amount of AgSO_4_ was controlled to be 1%, 3%, 5% and 7% to CoCl_2_·6H_2_O in molar ratio. The mixture solution was transferred into a stainless Teflon-lined autoclave and heated at 180 °C for 12 h. After the autoclave was cooled to room temperature, the product was thoroughly washed with DI water and ethanol, and dried at 80 °C. Undoped CoS was synthesized *via* the same way without adding AgSO_4_.

### 2.2. Electrode preparation

CoS or doped CoS is then coated on a FTO substrate according to a widely used CE preparation method.^32,33^ To prepare the CE for DSSCs, 0.2 g of the obtained nanopowders were suspended in 2 mL ethanol by sonication and magnetic stirring; then 0.86 mL terpineol and 1.1 mL ethyl cellulose in ethanol (10 wt%) were dipped into the mixture solution one by one, followed by again stirring and sonication. The resulting paste was coated onto the FTO glass (Sigma-Aldrich, *R* = 7 Ω sq^−1^) *via* spin coating method at 4000 rpm for 30 s. Afterwards, the CEs were annealed at 450 °C in Ar for 30 min. Moreover, the commercial Pt CE purchased from Dalian HepatChroma Solar-Tech Co., Ltd was used as a reference.

TiO_2_ nanoparticle photoanodes were prepared by spin-casting a ∼160 nm TiO_2_ under layer and doctor-blading technique to form a 10 μm TiO_2_ nanocrystalline layer. Then, the TiO_2_ photoanodes were immersed into 0.05 M TiCl_4_ aqueous solution at 70 °C for 30 min. Subsequently, the photoanodes were calcined at 450 °C for 0.5 h in air. After cooling at room temperature, the TiO_2_ photoanodes were took out and immersed in a 0.50 mM ethanol solution of N719 dye (purchased from DYESOL LTD) for 24 h. Finally, the dye-sensitized TiO_2_ photoanodes were took out from dye solution and washed with anhydrous ethanol. The active area of photoanodes was ∼0.25 cm^2^ (0.5 cm × 0.5 cm).

### 2.3. Fabrication of DSSCs

Each DSSC device was fabricated by combining a dye-sensitized TiO_2_ photoanode and a CE sandwiched with I_3_^−^/I^−^ based liquid electrolyte. The whole assembled arrangement was clamped. The liquid electrolyte was prepared by dissolving 10 mM of LiI, 1 mM of I_2_, and 0.1 mM of LiClO_4_ in acetonitrile.

### 2.4. Characterization and measurements

The composition of the CoS and Ag-doped CoS powders were detected by inductively coupled plasma-atomic emission spectra (ICP-AES). The result displays that the real atomic ratio of Co : S is nearly 1 : 1. The crystallographic structure was characterized by X-ray diffraction (XRD) on an X-ray powder diffractometer (Rigaku SmartLab9, Japan) using Cu Kα radiation (*λ* = 1.5406 Å). The morphology of nanopowders was characterized by using a scanning electron microscopy (SEM, Zeiss Supra 35VP, Berlin, Germany). High-resolution transmission electron microscopy (HRTEM) images of the Ag-doped CoS were acquired using a JEOL HRTEM (JEM-1400 electron microscope) with an acceleration voltage of 120 kV. The chemical states were analyzed *via* X-ray photoelectron spectroscopy (XPS) using a Thermo-ESCALAB 250XI (Thermo, USA) instrument with non-monochromated Al Kα 1486.6 eV radiation.

Cyclic voltammetry (CV) and electrochemical impedance spectra (EIS) measurements were conducted with each CE on a conventional Electrochemical Workstation (CHI600E, Shanghai Chenhua Co.). Cyclic voltammetry (CV) plots were recorded at a scan rate of 50 mV s^−1^ from −0.4 to 1.2 V in a three electrode setup: a FTO coated with CoS or Ag-doped CoS served as the working electrode, a Pt electrode served as the working electrode and an Ag/AgCl electrode served as reference electrode respectively. The diluted electrolyte for CV consisted of 10 mM LiI + 1 mM I_2_ + 100 mM LiClO_4_ in acetonitrile. The electrochemical impedance spectra (EIS) were carried out in the frequency range of 10^−2^ Hz to 10^6^ Hz in a two-electrode system (CE/electrolyte/CE). The magnitude of the alternative signal was 5 mV. The Tafel measurement was applied in the potential range of −1 V to +1 V. The current density–voltage (*J*–*V*) curves of the assembled DSSCs were measured on an Electrochemical Workstation (CHI600E, Shanghai Chenhua Co.) under simulated AM 1.5 sunlight at 100 mW cm^−2^ irradiance generated by a solar light simulator (Xe Lamp Oriel Sol3A™ Class AAA Solar Simulators 94023A, USA). Open Circuit Voltage Decay (OCVD) curves of DSSCs were recorded by a Electrochemical Workstation (CHI760D, Shanghai Chenhua Co.).

## 3. Results and discussion

The Ag amount in the Ag-doped CoS was characterized by inductively coupled plasma (ICP) analysis and the results are shown in Table 1. It can be concluded that the amount of Ag doping could be controlled by varying the amount of AgSO_4_ added into the reaction in this approach.

**Table 1:** Ag amount of the Ag–CoS composites by ICP analysis

Samples	The amount of AgSO_4_	Ag atomic content measured by ICP
CoS	—	—
1% Ag-doped CoS	1%	0.7%
3% Ag-doped CoS	3%	2.6%
5% Ag-doped CoS	5%	3.9%
7% Ag-doped CoS	7%	5.8%

**Fig. 1:**
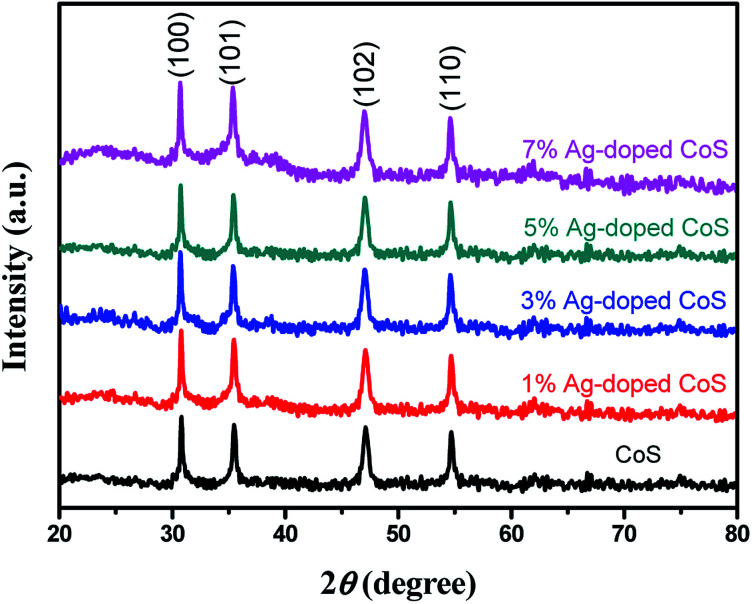
XRD patterns of undoped CoS and Ag-doped CoS samples with Ag contents of 1%, 3%, 5% and 7%.

Fig. 1 presents XRD patterns of the pure undoped CoS and Ag-doped CoS with varied Ag concentrations. The diffraction peaks of undoped CoS at 2*θ* = 30.6°, 35.4°, 47.0° and 54.5° corresponding to the planes of (100), (101), (102) and (110) can be indexed to hexagonal phase CoS (JCPDS card no. 65-3418). By doping Ag into CoS, no additional peaks were found, indicating that Ag doping does not change the crystal structure of CoS and no new phase is formed. However, a tiny shift towards the higher angles occurs with the increased Ag content. For instance, the 2*θ* angle for the (102) peak decreases from 47.1° for undoped CoS to 47.0° for 5% Ag-doped CoS. Such peak position changes are ascribed to a certain amount of Co^2+^ ions (radius = 65 pm) being substituted by larger Ag^+^ ions (radius = 115 pm), resulting in the expansion of the lattice parameter in an axis.^34,35^

**Fig. 2:**
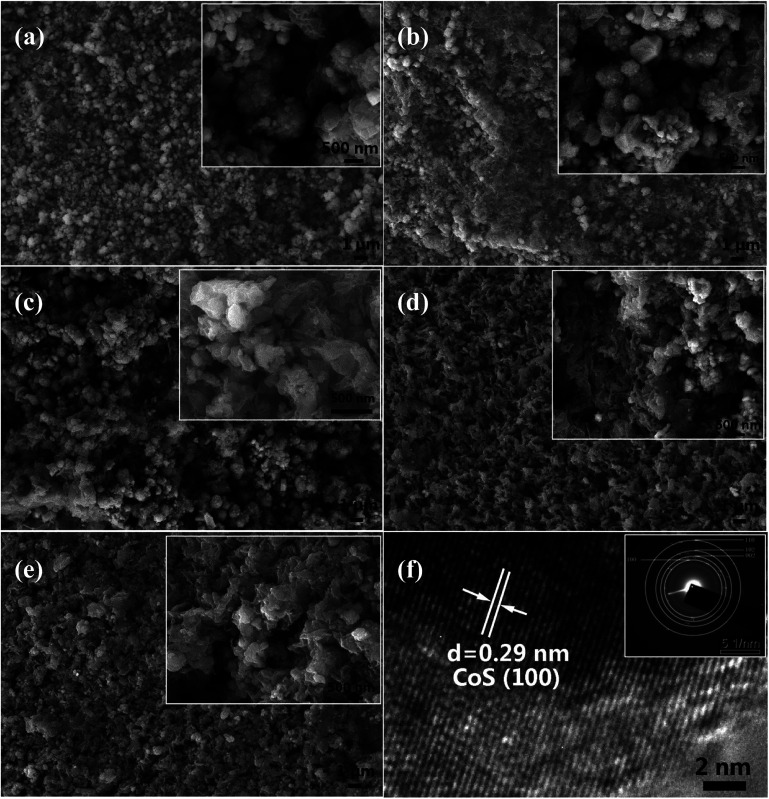
SEM images of (a) undoped CoS; (b) 1% (c) 3% (d) 5% (e) 7% Ag doped CoS. (f) HRTEM and SAED pattern (the inset) of the 5% Ag-doped CoS.

The field emission scanning electron microscopic (FESEM) images of the films of undoped CoS and Ag-doped CoS with varied Ag concentrations are shown in Fig. 2(a–e), respectively. Agglomerated nanoparticles and nanosheets co-exist in all the samples. It can be clearly seen that the obtained undoped CoS and 1% Ag doped CoS mostly exhibit agglomerated nanoparticles morphology. FESEM images also reveal that with the increase of Ag concentration the number of nanoparticles decreases, possibly because element doping with larger ion radius may suppress the nucleation of nanoparticles. In DSSCs, the morphologies of CE materials have an important effect on the catalytic reactions because of the catalytic reactions occur on the surface of the CEs. In this study, the nanosheets architecture facilitates the transfer of charge carriers from their surface to the electrolyte. To further identify the crystallinity of the Ag-doped CoS, HRTEM and SAED are conducted on 5% Ag-doped CoS (Fig. 2(f)). The HRTEM image exhibits lattice fringes with spacings of 0.29 nm, corresponding to the (100) plane of hexagonal CoS, and a SAED pattern in the inset of Fig. 2(f) is indexed to a hexagonal CoS phase with a few characteristic (100), (110), (102) and (002) planes. The results are consistent with the XRD results reported in this paper earlier.

**Fig. 3:**
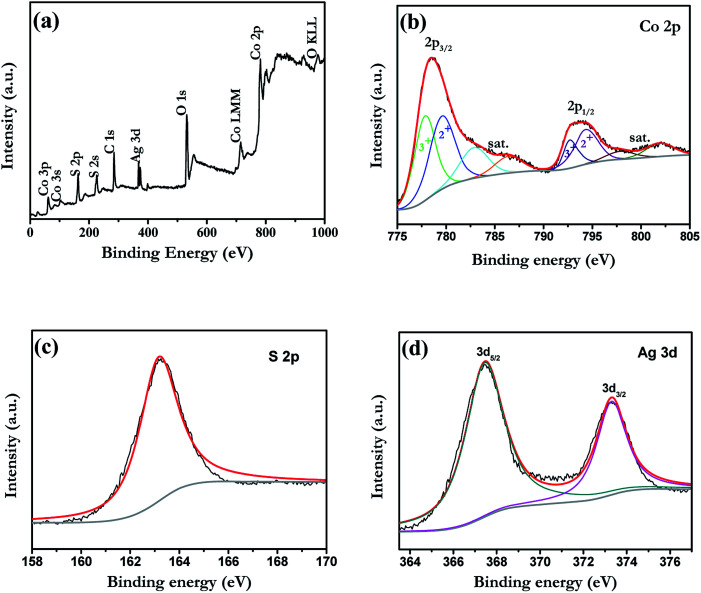
XPS spectra of the as-prepared 5% Ag-doped CoS: (a) survey; (b) Co 2p; (c) S 2p; (d) Ag 3d.

In order to reveal the details on the chemical states of Ag-doped CoS, X-ray photo electron spectroscopy (XPS) analysis was performed (Fig. 3). Take 5% Ag-doped CoS as an example, the peaks of Co, S and Ag can be observed in the survey spectrum. As shown in Fig. 3(b), the content of the Co 2p spectrum was quite complex owing to the presence of various species at surface level. After fitting, the Co 2p_2/3_ spectrum has binding energies at 777.9 and 780.1 eV that can be attributed to sulfided Co–S.^36,37^ The peaks between 792.0 and 803.0 eV belong to the Co 2p_1/2_ signals of their Co 2p_3/2_ counterparts and the satellite signal.^38^ The S 2p peak centered at 163.2 eV is typical for a Co–S bond.^39^ Therefore, the major phase of the cobalt sulfide (Co_*x*_S_*y*_) is CoS, while small amount of Co_*x*_S_*y*_ (*x* = 1, 2…4; *y* = 1, 2, 3…9) compounds are also formed during the preparation process of counter electrodes.^40^Fig. 3(d) shows the XPS Ag 3d core level spectrum. It can be fit by two peaks at 373.3 eV and 367.5 eV for Ag 3d_3/2_ and 3d_5/2_, respectively, with a spin–orbital splitting of 5.8 eV, which can be considered as the standard reference XPS spectrum of Ag(i).^41^

**Fig. 4:**
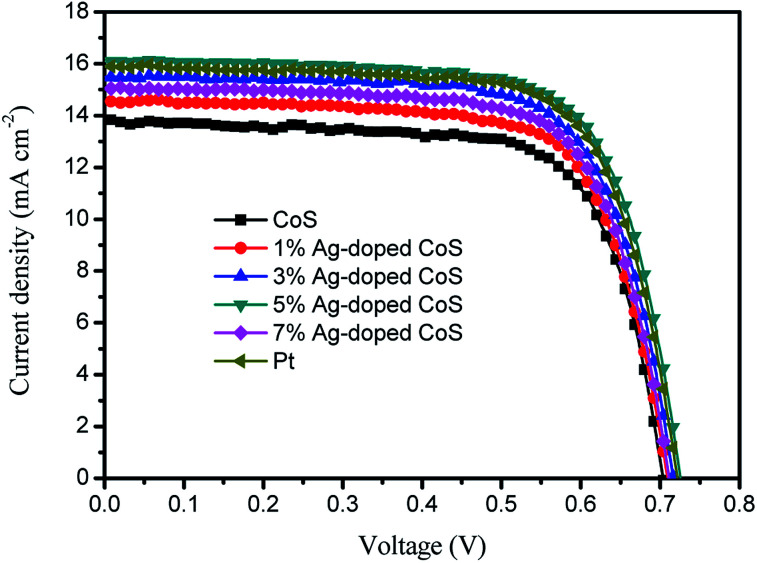
Photocurrent density–voltage (*J*–*V*) characteristics of DSSCs with different CEs, measured under the AM 1.5 illumination (100 mW cm^−2^).

To investigate the photovoltaic properties of the DSSCs with the undoped and Ag-doped CoS based CEs, the photocurrent-density–voltage (*J*–*V*) curves are shown in Fig. 4. The corresponding photovoltaic parameters are summarized in Table 2. A maximum PCE of 6.93% with *J*_sc_ of 13.84 mA cm^−2^ and *V*_oc_ of 0.704 mV was achieved with the DSSC with the undoped CoS CE. The PCEs are 7.35%, 7.89%, 8.35%, and 7.61% for DSSCs with the 1%, 3%, 5% and 7% Ag-doped CoS CEs, respectively, indicating a markedly increased PCE with increasing Ag doping. It is expected that the efficiency of the DSSC based on doped CoS initially increases with the increased Ag content and reaches a maximum point of 8.70% when the Ag amount is 5%, which is higher than those of DSSCs based on undoped CoS (6.93%) by 20.5% and Pt (8.17%) by 2.2%, though the DSSC PCE decreases when the content of Ag impurity is further increased after 5%. The enhanced PCE can be mainly derived from the increasing electrical conductivity and electrocatalytic activity resulting from Ag-doping atoms. The doped Ag ions introduce large amount of holes and induce the increasing charge carrier density for electron conduction. Meanwhile, CoS experiences reduced particle size after Ag doping, which increases the amount of grain boundaries and thus provides a fast ion diffusion pathway. Hence, the electrical and ionic conductivity are enhanced simultaneously. At the same time, doping with Ag ions increases the surface to volume ratio of the CE, leading to more active sites for redox reaction and superior electrocatalytic activity.

**Table 2:** Output photovoltaic characteristics of the DSSCs employing different CEs

CEs	*V*_oc_ (V)	*J*_sc_ (mA cm^−2^)	FF	PCE (%)
CoS	0.704	13.84	0.711	6.93
1% Ag-doped CoS	0.710	14.53	0.713	7.35
3% Ag-doped CoS	0.716	15.48	0.711	7.89
5% Ag-doped CoS	0.726	16.13	0.713	8.35
7% Ag-doped CoS	0.711	15.03	0.713	7.61
Pt	0.722	15.89	0.712	8.17

**Fig. 5:**
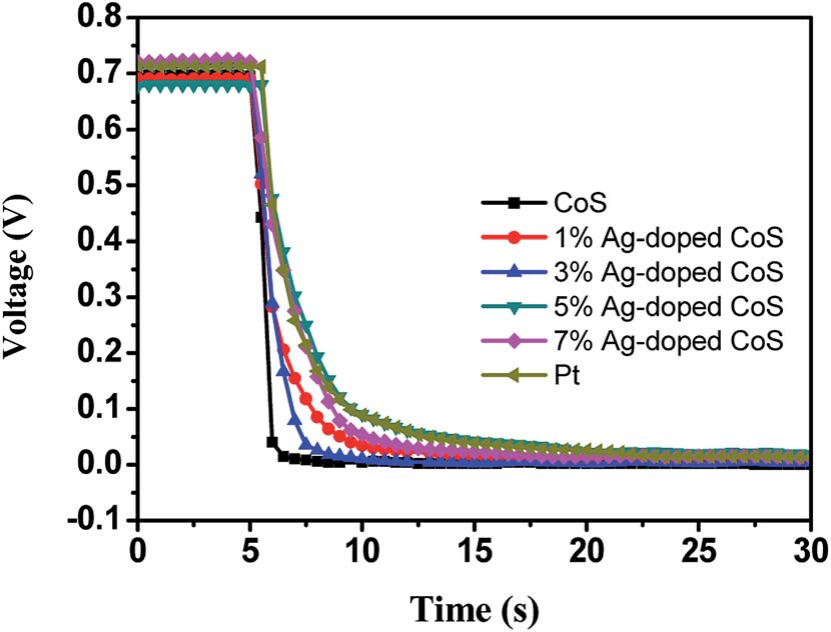
OCVD curves observed with different CEs.

Fig. 5 shows the OCVD curves of the DSSC based on pure CoS and Ag-doped CoS films as a CE, which demonstrates the electron lifetime containing a wealth of information on the electron recombination process in a DSSC. During OCVD measurement, DSSCs were illuminated and the subsequent photovoltage decay after interrupting the illumination was monitored. The slower decay obtained for the DSSC fabricated using 5% Ag-doped CoS and Pt as the CE was the best among our experimental results. This may be attributed to the adequate Ag dopants in CoS leading to a lower rate of electron loss, indicating a higher electron lifetime for the DSSCs. It can be seen clearly that the OCVD response of DSSC with the bare CoS CE was significantly faster than other five curves, which indicates a higher recombination rate and shorter electron lifetime.

**Fig. 6:**
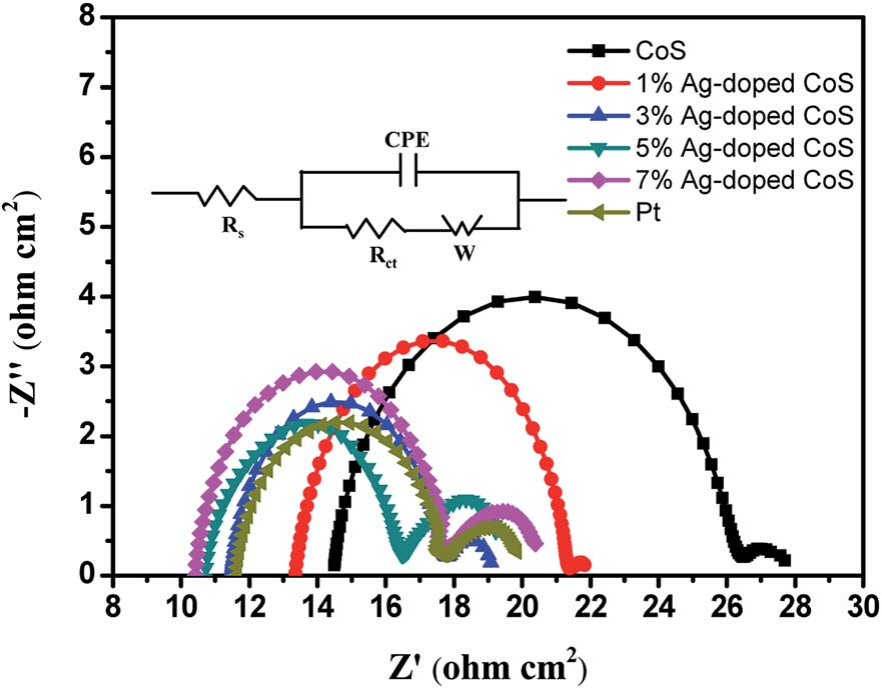
Nyquist plots of the symmetrical cells based on different CEs; inset: the equivalent circuit.

The electrochemical characteristics of the undoped and Ag-doped CoS CEs were evaluated by electrochemical impedance spectroscopy (EIS) and cyclic voltammetry (CV) tests. Fig. 6 shows the EIS in Nyquist plots, and the experimental results (Table 3) were fitted using the equivalent circuit in the inset of Fig. 6. The intercepts with the real axis in the high frequency range represent the *R*_s_ (overall ohmic series resistance) values, including the bulk resistance of the CE materials, the FTO substrate and the contact resistance.^42^ The *R*_s_ of undoped CoS CE is 14.49 Ω cm^2^. The *R*_s_ value is reduced to 13.38 Ω cm^2^ after being doped with 1% Ag, indicating an evident increase in electrical conductivity. Though undoped CoS is an n-type semiconductor due to its intrinsic impurity,^43^ a large number of holes are introduced with Ag doping, and Ag-doped CoS is directly converted to a p-type material, resulting in a markedly increased charge carrier density. With the increased Ag content, the charge carrier density is further increased and therefore *R*_s_ keeps decreasing. *R*_ct_ value is widely used to estimate the electron exchange ability between the CE and the liquid electrolyte and examine the electrocatalytic activity of the CEs.^44,45^ A lower *R*_ct_ value means a higher charge transfer rate at CE/electrolyte interface and therefore higher electrocatalytic reduction of I_3_^−^.^46^ The *R*_ct_ keeps decreasing as Ag content increases until 5% (5.76 Ω cm^2^), reaching a lower value than that of Pt (6.10 Ω cm^2^), as we have shown in Table 3, indicating an evident improvement in the electrocatalytic activity for iodide/triiodide redox reaction because of the reduced agglomerated nanoparticles quantity and larger surface to volume ratio caused by Ag doping. However, the *R*_ct_ of the 7% Ag-doped CoS CE increases to 7.32 Ω cm^2^, since its catalytic activity is depressed probably due to a serious lattice disorder. Overall, Ag doping significantly enhances the electrical conductivity of the CoS CE; optimized Ag-doping level can also increase the electrocatalytic activity of the CE significantly, which results in a remarkable increase of PCE.

**Table 3:** Electrochemical parameters obtained from CV and EIS characterizations

CEs	*E*_pp_ (V)	*J*_Red-1_ (mA cm^−2^)	*R*_s_ (Ω cm^2^)	*R*_ct_ (Ω cm^2^)
CoS	0.472	4.01	14.49	12.03
1% Ag-doped CoS	0.452	4.81	13.38	8.03
3% Ag-doped CoS	0.446	5.15	11.50	6.28
5% Ag-doped CoS	0.422	5.56	10.73	5.76
7% Ag-doped CoS	0.450	5.01	10.44	7.32
Pt	0.427	5.38	11.64	6.10

**Fig. 7:**
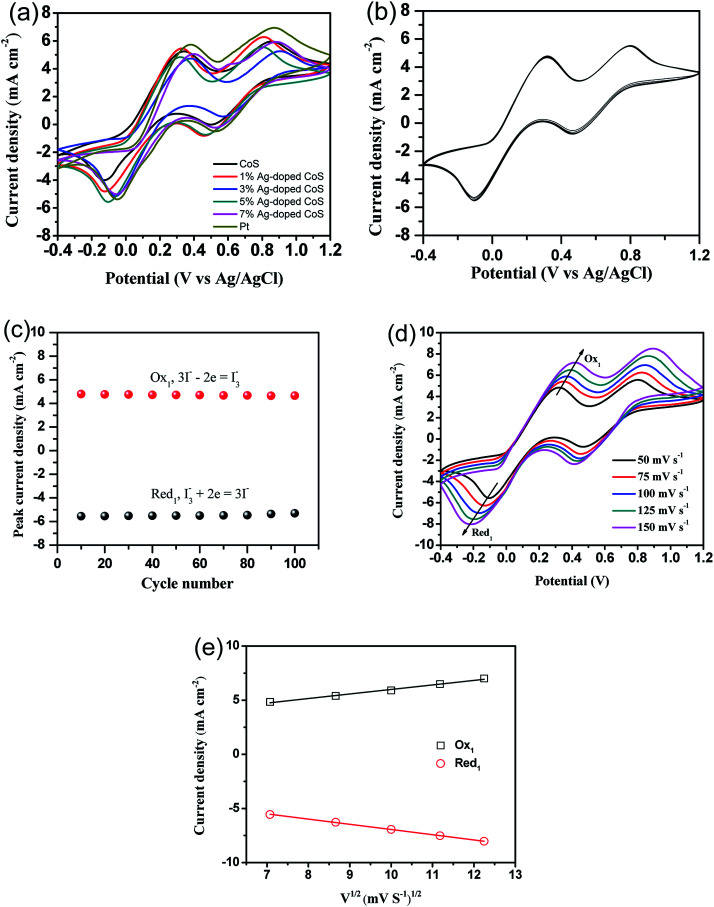
(a) CV curves of different CEs at a scan rate of 50 mV s^−1^, (b) the 100-stacking CV curves from 5% Ag doped CoS CE at a scan rate of 50 mV s^−1^, and (c) the peak current density stability as a function of cycle number. (d) CVs for 5% Ag doped CoS electrode recorded at different scan rates of 50, 75, 100, 125 and 150 mV s^−1^ and (e) the relationship between redox current density and square root of scan rates of CVs for 5% Ag doped CoS CE.

To further investigate the electrochemical catalytic activities of these CEs, CV was performed for a three electrode system (Fig. 7). For all the CEs, two pairs of oxidization and reduction peaks are presented in the CV curves. The relative low-potential peaks correspond to the reaction in eqn (1), while high-potential peaks correspond to the reaction in eqn (2).^5,33^

1 I_3_^−^ + 2e = 3I^−^

2 3I_2_ + 2e = 2I_3_^−^

The electrocatalytic activity of the CEs for the reduction of triiodide can be evaluated according to the peak-to-peak voltage separation (*E*_pp_), which is negatively correlated with the standard electrochemical rate constant of a redox reaction. The *E*_pp_ value of the undoped CoS CE (0.472 V) is significantly higher than the *E*_pp_ value of the Pt CE (0.427 V), suggesting a higher over potential for reduction of I_3_^−^ to I^−^. The *E*_pp_ keeps decreasing until the doping content reaches 5% (0.422 V), thus indicating an improved electrocatalytic activity. But the *E*_pp_ increases to 0.450 V when the Ag-doping concentration is increased to 7%. The value of *E*_pp_ decreases in the order of undoped CoS (0.472 V) > 1% Ag-doped CoS (0.452 V) > 7% Ag-doped CoS (0.450 V) > 3% Ag-doped CoS (0.446 V) > Pt (0.427 V) > 5% Ag-doped CoS (0.422 V). Overall, the 5% Ag-doped CoS electrode shows the most narrowed *E*_pp_ value of 0.422 V, indicating that this CE shows the best electrocatalytic activity compared with other CEs. In order to investigate the stability of the CE in the liquid electrolyte, CV measurements have been performed on the CE based on 5% Ag-doped CoS at a scan rate of 50 mV s^−1^ for 100 cycles (see Fig. 7(b)). As shown in Fig. 7(c), no apparent decrease in current density during cycling has been observed, indicating that this CE exhibits a good electrochemical stability as the CE for DSSCs. In addition, Fig. 7(d) shows CVs of the I^−^/I_3_^−^ redox pair using the 5% Ag doped CoS electrode with different scan rates of 50, 75, 100, 125 and 150 mV s^−1^. The CVs exhibit a regularly outward extension of all peaks with the increasement of scan rates. From Fig. 7(e), it is obvious that the anodic and cathodic peaks current density both show good linear with the square root of scan rates, indicating the diffusion of I^−^ controls the redox reaction on the surface of the CEs and there is no specific interaction between the prepared CE and I^−^/I_3_^−^ redox pair.^47^

**Fig. 8:**
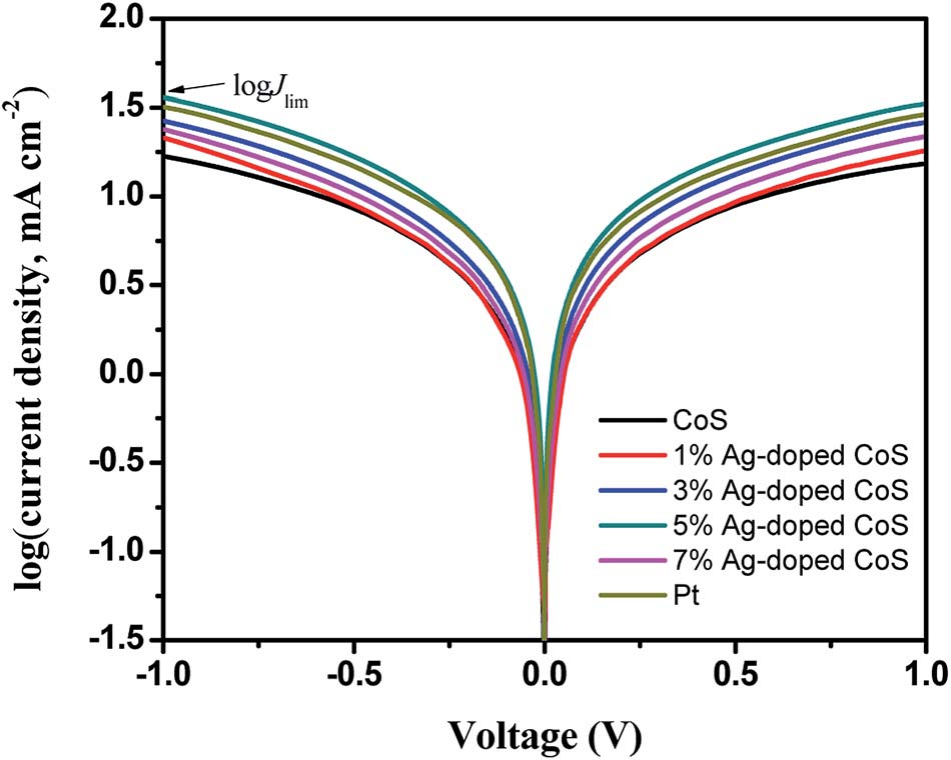
Tafel-polarization curves of different CEs.

In order to assess the contribution of mass diffusion rate to the improvement of PCE, Tafel-polarization measurements are conducted to estimate the anodic and cathodic steady-state polarization diffusion-limited current (*J*_lim_). As seen in Fig. 8, the limiting current density plateaus of all the cells are well developed, indicating that they have reached the diffusion-limiting region in the given potential range.^48^ Furthermore, the ionic diffusion coefficient of the triiodide species, which was determined by the diffusion of ionic carriers between the two electrodes, was directly proportional to the limiting current density *J*_lim_.^49,50^ The cell based on pure CoS CEs exhibits the lowest current density, indicating its lowest ion diffusion rate. In the case of Ag-doped CoS CEs, the current density keeps increasing with the increasing Ag content, suggesting an improved ionic conductivity. Obviously, the ion diffusion rate increased in the order of undoped CoS (16.86 mA cm^−2^) < 1% Ag-doped CoS (21.46 mA cm^−2^) < 7% Ag-doped CoS (23.95 mA cm^−2^) < 3% Ag-doped CoS (26.72 mA cm^−2^) < Pt (31.99 mA cm^−2^) < 5% Ag-doped CoS (36.17 mA cm^−2^), which is in good agreement with the *J*_Red-1_ trend in CV results. Therefore, the Ag-doped CoS CEs profit from the accelerated mass transfer rates that results in an increased PCE.

## 4. Conclusions

This study introduces a facile one-step route to fabricate low-cost Ag-doped CoS films for application as counter electrodes in next-generation DSSCs. The obtained Ag-doped CoS CEs exhibit significantly enhanced electrocatalytic activity and mixed ionic–electronic conductivity compared to pure CoS CE. The Ag-doping amount can be easily adjusted to optimize the DSSC performance, and it is found that the DSSC with the 5% Ag-doped CoS CE achieves the highest PCE of 8.35%, exceeding those of DSSCs based on the Pt CE (8.17%) by 2.2% and undoped CoS (6.93%) by 20.5%. Such an improved DSSC efficiency is attributed to the effect of Ag-doping on structural and chemical properties of the CoS-based CEs. The results of this study indicate that the low-cost Ag-doped CoS CE is a promising alternative to the costly Pt CE in DSSCs.

## Conflicts of interest

The authors declare no conflict of interest.

## Supplementary Material

RA-008-C8RA02765J-s001
