# Supplementary material for: High-performance dye-sensitized solar cells using Ag-doped CoS counter electrodes
Source: RSC Adv. 2018 May 22;8(34):18792–9. doi: 10.1039/c8ra02765j (PMC9080620; doi:10.1039/c8ra02765j)
Supplement: RA-008-C8RA02765J-s001 [file RA-008-C8RA02765J-s001.pdf]

Electronic Supplementary Material (ESI) for RSC Advances.
This journal is © The Royal Society of Chemistry 2018

Supporting Information

High-performance dye-sensitized solar cells using Ag-doped CoS counter electrodes

Guoce Zhuang, Huiling Liu, Xiaobo Chen

School of New Energy and Electronic Engineering, Yancheng Teachers University, Yancheng, 224051, PR China

Table S1. Comparison of the power conversion efficiency between the reported CoS based DSSCs and the present work.

**CEs**	***η* (%)**	**Ref.[a]**
porous CoS	6.33%	S1
CoS acicular nanorod arrays	7.67%	S2
CoS nanoparticles	8.1%	S3
honeycomb-like CoS	7.72%	S4
CoS-Graphene Composite	6.31%	S5
CoS nanosheet arrays	6.39%	S6
CoS/graphene composite	7.08%	S7
Nickel doped cobalt sulfide	5.50%	S8
mixed-phase cobalt sulfide	7.2 %	S9
PANi-CoS	8.55%	S10
CoS nanosheets-coupled graphene quantum dots	7.30%	S11
PAN/CoS nanocomposite	7.41%	S12
5% Ag-doped CoS	8.35%	This work

[a]Reference:

S1. Lin, J. Y., Liao, J. H., & Chou, S. W. (2011). Cathodic electrodeposition of highly porous cobalt sulfide counter electrodes for dye-sensitized solar cells. Electrochimica Acta, 56(24), 8818-8826.

S2. Kung, C. W., Chen, H. W., Lin, C. Y., Huang, K. C., Vittal, R., & Ho, K. C. (2012). CoS acicular nanorod arrays for the counter electrode of an efficient dye-sensitized solar cell. ACS nano, 6(8), 7016-7025.

S3. Hsu, S. H., Li, C. T., Chien, H. T., Salunkhe, R. R., Suzuki, N., Yamauchi, Y., ... & Wu, K. C. W. (2014). Platinum-free counter electrode comprised of metal-organic-framework (MOF)-derived cobalt sulfide nanoparticles for efficient dye-sensitized solar cells (DSSCs). Scientific reports, 4, 6983.

S4. Huo, J., Zheng, M., Tu, Y., Wu, J., Hu, L., & Dai, S. (2015). A high performance cobalt sulfide counter electrode for dye-sensitized solar cells. Electrochimica Acta, 159, 166-173.

S5. Wang, F., Wu, C., Tan, Y., Jin, T., Chi, B., Pu, J., & Jian, L. (2015). CoS-Graphene composite counter electrode for high performance dye-sensitized solar cell. Journal of nanoscience and

nanotechnology, 15(2), 1180-1187.
S6. Tai, S. Y., Chang, C. F., Liu, W. C., Liao, J. H., & Lin, J. Y. (2013). Optically transparent counter electrode for dye-sensitized solar cells based on cobalt sulfide nanosheet arrays. Electrochimica Acta, 107, 66-70..
S7. Wang, G., Zhang, J., Kuang, S., Liu, S., & Zhuo, S. (2014). The production of cobalt sulfide/graphene composite for use as a low-cost counter-electrode material in dye-sensitized solar cells. Journal of Power Sources, 269, 473-478.
S8. Kim, H. J., Kim, C. W., Punnoose, D., Gopi, C. V., Kim, S. K., Prabakar, K., & Rao, S. S. (2015). Nickel doped cobalt sulfide as a high performance counter electrode for dye-sensitized solar cells. Applied Surface Science, 328, 78-85.
S9. Luo, Y., Shen, J., Cheng, R., Chen, X., Chen, Y., Sun, Z., & Huang, S. (2015). Facile synthesis of mixed-phase cobalt sulfide counter electrodes for efficient dye sensitized solar cells. Journal of Materials Science: Materials in Electronics, 26(1), 42-48.
S10. Yang, P., Duan, J., & Tang, Q. (2015). Cobalt sulfide decorated polyaniline complex counter electrodes for efficient dye-sensitized solar cells. Electrochimica Acta, 184, 64-69.
S11. Yu, C., Liu, Z., Chen, Y., Meng, X., Li, M., & Qiu, J. (2016). CoS nanosheets-coupled graphene quantum dots architectures as a binder-free counter electrode for high-performance DSSCs. Science China Materials, 59(2), 104-111.
S12. Murugadoss, V., Arunachalam, S., Elayappan, V., & Angaiah, S. (2018). Development of electrospun PAN/CoS nanocomposite membrane electrolyte for high-performance DSSC. Ionics, 1-10.
